# Genome-Wide Association Analysis with Gray Matter Volume as a Quantitative Phenotype in First-Episode Treatment-Naïve Patients with Schizophrenia

**DOI:** 10.1371/journal.pone.0075083

**Published:** 2013-09-24

**Authors:** Qiang Wang, Bo Xiang, Wei Deng, Junyao Wu, Mingli Li, Xiaohong Ma, Yingcheng Wang, Lijun Jiang, Grainne McAlonan, Siew E. Chua, Pak C. Sham, Xun Hu, Tao Li

**Affiliations:** 1 Mental Health Center, West China Hospital, Sichuan University, Chengdu, Sichuan, P R China; 2 State Key Laboratory of Biotherapy, Psychiatric Laboratory, West China Hospital, Sichuan University, Chengdu, Sichuan, P R China; 3 Department of Psychiatry, University of Hong Kong, Hong Kong, P R China; 4 Department of Forensic and Neurodevelopmental Sciences, Institute of Psychiatry, King’s College London, London, United Kingdom; 5 Biobank, West China Hospital, Sichuan University, Chengdu, Sichuan, P R China; Kunming Institute of Zoology, Chinese Academy of Sciences, China

## Abstract

Reduced Gray matter (GM) volume is a core feature of schizophrenia. Mapping genes that is associated with the heritable disease-related phenotypes may be conducive to elucidate the pathogenesis of schizophrenia. This study aims to identify the common genetic variants that underlie the deficits of GM volume in schizophrenia. High-resolution T1 images and whole genome genotyping data were obtained from 74 first-episode treatment-naïve patients with schizophrenia and 51 healthy controls in the Mental Health Centre of the West China Hospital, Sichuan University. All participants were scanned using a 3T MR imaging system and were genotyped using the HumanHap660 Bead Array. Reduced GM volumes in three brain areas including left hOC3v in the collateral sulcus of visual cortex (hOC3vL), left cerebellar vermis lobule 10 (vermisL10) and right cerebellar vermis lobule 10 (vermisR10) were found in patients with schizophrenia. There was a group by genotype interaction when genotypes from genome-wide scan were subsequently considered in the case-control analyses. SNPs from three genes or chromosomal regions (TBXAS1, PIK3C2G and HS3ST5) were identified to predict the changes of GM volume in hOC3vL, vermisL10 and vermisR10. These results also highlighted the usefulness of endophenotype in exploring the pathogenesis of neuropsychiatric diseases such as schizophrenia although further independent replication studies are needed in the future.

## Introduction

Schizophrenia is one of the leading causes of mental disability, which affects about 1% of the population worldwide [1]. It has been suggested that genetic factors plays an important role in the pathophysiology of schizophrenia by many convincing studies [2]. Indeed, the heritability of schizophrenia is as high as 80% [3]. However, it is challenging to identify genes involved in the pathogenesis of schizophrenia due to its complex model of inheritance and the unknown pathophysiology of the disorder. Furthermore, phenotypic heterogeneity, such as various clinical presentation and duration of illness, complicated the genetic study in schizophrenia.

Recently, a number of large-scale genome-wide association studies (GWAs) using the case–control design have identified some genetic variants associated with schizophrenia (http://www.genome.gov/page.cfm?pageid=26525384&clearquery=1#result_table). Of these previous studies, the latest Psychiatric Genomics Consortium (PGC) identified 61 common variants in liability to schizophrenia. Two studies in Han Chinese population suggested some common variants involved in susceptibility of schizophrenia [4,5], though there were no overlapping SNPs detected from the two studies. One of possible reasons may be that the heterogeneity of clinical manifestations such as the severity of disease and more subtle characteristics were ignored in large according to the clinical diagnostic categories. The current diagnosis for schizophrenia mainly depends on subjective and qualitative information. In case-control design of GWAs, it is also possible that a small proportion of current healthy controls have actually harbored disease because of the long clinical silent prodromal phase. On the other hand, although the GWAs have successfully identified some risk genes of complex diseases including diabetes, macular degeneration, Crohn disease, Alzheimer disease, and Parkinson disease [6], it is a great challenge to have enough sample sizes to obtain satisfied statistical power, and to control population stratification which is due to the combination of samples from multiple ethnic backgrounds. The unsatisfied statistical power due to small sample size can be improved by using a quantitative trait (QT) analysis [7]. Compared to a simple dichotomous classification of diagnosis, QT related to studied disorder is an objective and informative measurement and could be used as endophenotypes or intermediate phenotypes in genetic analysis. Endophenotypes have been considered to be more proximal to the biological etiology of the disorder [8], and may provide an alternative strategy to explore the pathogenesis of complex genetic diseases such as schizophrenia.

The meta-analytic, twin and family studies showed that whole and regional GM volumes are highly heritable [9,10]. And GM volume abnormalities covary in a dose-dependent manner with risk for schizophrenia [11,12]. Thus it should be a good strategy to use GM volume as one of the endophenotypes in molecular studies of schizophrenia. Some of the previous studies have successfully performed candidate gene analyses using GM as QT [13-15], however, only 3 studies employed GWAs design for schizophrenia [7,16,17]. Moreover, QT-GWAs design in Chinese sample is still rare.

The aim of the present study is to identify the common genetic variants that underlie the deficits of GM volume in schizophrenia. We used the SPM Anatomy Toolbox [18-20] to obtain anatomical region of interests (ROIs) volume and to detect the brain areas with different ROIs volumes between patients with first-episode treatment-naïve schizophrenia and healthy controls. Subsequently, GM volumes in these brain areas were integrated into genetic data from GWAS analysis as QTs in order to identify novel susceptibility loci for schizophrenia.

## Materials and Methods

### Participants

The study was approved by the Ethics Committee of the West China Hospital of Sichuan University. All next of kin, carer takers or guardians consented on the behalf of participants to provided written informed consent for their participation. We used the following criteria to evaluate whether the participants had the capacity to consent: Firstly, patients have the ability to understand; Second, patients have the ability to know reason; Thirdly, patients have the ability to make rational decisions. If participants failed to fill out the consent form more than twice, their guardians were asked to fill out the consent on the behalf of patients. A total of 125 participants were recruited at the Mental Health Centre of the West China Hospital, Sichuan University, People Republic of China, including 74 first-episode patients with schizophrenia and 51 healthy controls. The Structured Clinical Interview for DSM-IV (Diagnostic and Statistical Manual of Mental Disorders, fourth edition) – Patient Version (SCID-P) was used and patients fulfilling the diagnostic criteria for schizophrenia as specified in DSM-IV were included. The interviews were performed and the diagnoses of schizophrenia were confirmed by an attending psychiatrist and a trained interviewer. Healthy controls were recruited from the local area by poster advertisement and they were screened for the lifetime absence of psychiatric illnesses by the SCID non-patient version (SCID-NP). All healthy controls were interviewed to assure that there was no history of psychiatric illness in their first-degree relatives. All patients with schizophrenia have been followed up for at least 6 months in order to confirm the diagnosis of schizophrenia. All participants are Han Chinese from Sichuan province of China.

### Imaging acquisition

One patient and one control were excluded from subsequent analysis because of poor images’ quality. Finally, data were acquired for the 125 participants undergoing MRI scans in the Department of Radiology at West China Hospital, Sichuan university with a 3T MR imaging system (EXCITE, General Electric, Milwaukee, USA) which has an eight-channel phase array head coil. High-resolution T1 images were obtained by the 3D spoiled gradient echo sequence (SPGR) from all participants. The protocols used were as follows: TR=8.5ms; TE=3.93ms; ﬂip angle=12°; thickness of slice=1 mm; single shot; field of view (FOV) =24cm×24cm; matrix =256×256; size of voxel=0.47×0.47×1 mm^3^. In total, 156 slices of axial images were collected from each brain.

### Image preprocessing

The DICOM format data were collected from magnetic resonance scanning and then transformed using MRIcro software [41]. We used the non-parametric non-uniformity intensity normalization (N3) in the MINC software package (http://wiki.bic.mni.mcgill.ca/index.php/MINC) to correct the intensity inhomogeneity of images by manually aligning on the AC-PC line. The Brain Extraction Tool (BET) in FSL software was used to remove non-brain tissues and structures in the images [42] and to obtain native images for further processing. In order to process the data, Statistical Parametric Mapping (SPM2 package, Wellcome Department of Imaging Neuroscience, London; http://www.fil.ion.ucl.ac.uk/spm) software and the optimized VBM method were implemented in Matlab 7.0. All the 125 participants underwent structural MRI scans (in native space) and the brain areas were segmented into GM, white matter (WM) and cerebrospinal ﬂuid (CSF). We obtained the GM images which were affine-normalized in the same stereotactic space (International Consortium for Brain Mapping) according to the GM template (gray. mnc). The normalization parameters were implemented to the original structural images. Then the tissue segmentations were applied to normalized images to obtain image of GM, WM and CSF. Specifically, the images were normalized and segmented by an integrated generative model (unified segmentation) [43] with the customized templates. Jacobian determinants derived from the spatial normalization step were applied to the segmented images to correct voxel signal intensity for volume displacement and to reflect the volume during normalization. Finally we used a 6-mm FWHM kernel to smooth each optimally normalized, segmented and modulated image. The SPM Anatomy Toolbox [18] was applied to obtain anatomical ROIs volumes.

## Statistical Analysis

### Imaging

In order to detect whether there were any differences in the volume of areas extracted from smoothed images by the SPM Anatomy Toolbox between schizophrenia and control individuals, we used SPSS16.0 statistical software to perform an ANOVA analysis to explore the differences of GM volume between schizophrenia and controls, age and gender were incorporated as covariates. Statistically signiﬁcant *p* value thresholds were set at<0.05 after Bonferroni correction.

### Genotyping and quality control

We obtained DNA with the standard isolation method from whole blood samples and performed genotyping using the HumanHap660 Bead Array. The quality of the SNP genotypes was assessed for genotyping rates across samples, minor allele frequency (MAF) and Hardy-Weinberg equilibrium (HWE) tests (only in controls). Participants with genotyping rate < 95% were excluded. The gender of each participant was confirmed with genotyping data on gender-specific loci provided by Illumina. For pairs of participants with identical genotypes, the member with the lower genotype call rate was excluded. Finally, 99393 SNPs were removed with more than 10% missing genotypes across samples, 95922 SNPs were removed with MAF<5% and 331 SNPs failed the HWE test in control subjects (i.e. *p*-value <10^-5^). Consequently a total of 464,219 SNPs passed the quality control and a mean call rate of 98.9% was obtained; 2 patients and 1 control failed gender check and were excluded from subsequently analysis. Finally, 74 patients and 51 controls passed quality control and were included in subsequent analysis.

### Genotype imputation

BEAGLE was employed to infer genotypes for untyped SNPs. We used the haplotypes from the HapMap project as the reference dataset for the genotype imputation, which include the phased genotypes of 45 subjects from the CHB panel of HapMap Phase 2, 41 subjects from the CHB panel and 83 subjects from the CHD panel of HapMap Phase 3. The phased genotypes were downloaded from http://hapmap.ncbi.nlm.nih.gov/downloads/phasing/?N=D. The tool IGG3 [44] (http://bioinfo.hku.hk/iggweb/) was used to integrate the phased genotypes into our GWAS genotype dataset and to export the integrated data in BEAGL input format. In imputation, the parameters of BEAGLE were set by default.

### Correction for population stratification

Population stratification was detected by using EIGENSTART [45], which employs principal components analysis (PCA) to capture hidden population structure in GWAS data. Prior to the PCA, chromosomal regions of long-range linkage disequilibrium (LD) [46] were removed and the SNPs were pruned so that the retained neighboring SNPs were in weak LD with each other (with PLINK command -- indep-pairwise 50 10 0.5). PCA was performed on the remaining set of 189,398 SNPs. The top 10 principal components (PCs) were extracted for subsequent QT analyses as covariates (See Figure S1).

### QT analysis

**Figure 1:**
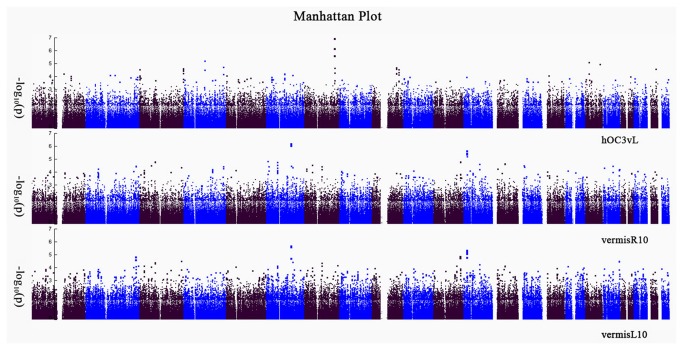
Manhattan plot for the Han Chinese genome-wide association study (GWAS) on schizophrenia using brain structure measures as a quantitative phenotype (Note: the points with red mean their P values >5x10^-6^).

The volumes of hOC3vL, vermisL10 and vermisR10 (see Figure S2), which were detected to be different between the patients and controls, were used as QTs in separate regression analyses. The 125 individuals who passed both imaging and genotyping quality control were included in QT analyses. The statistical model was based on comparing the differential effects of SNP association by diagnosis on the brain imaging QT. Out of the 4 possible models (additive, codominant, dominant and recessive) the linear model with interactions between diagnosis and SNPs implements the additive model that generally reflects the additive contribution to risks for complex diseases [47]. For QT analyses, we implemented the PLINK program with linear model function (--linear) [48] (http://pngu.mgh.harvard.edu/~purcell/plink/), in which each QT was regressed on interactive effects between diagnosis and an amount of minor alleles of the 464,219 SNPs having passed quality control. The covariates (age and gender) were included in the linear model. Meanwhile, the top 10 PCs were included as covariates in QT analyses to control potential population stratification effects. In these models, we used an ordinal variable (controls =1, schizophrenia=2) to reflect the disease status. On the other hand, since in the initial analyses of interaction term model, there were a large number of variables analyzed and the complexity of the model was applied, we chose the *p* value threshold ≤ 10^-6^ to keep consistent with WTCCC recommendations [6]. Meanwhile, we employed an even more conservative regulation that required to detect at least 2 SNPs with the *p* value <10^-6^ within a gene or intergenic region for each QT analysis. The results were shown in Manhattan plots and Quantile–quantile plots (See Figure 1 and Figure S3).

### Set-based analysis

Set-based analyses were used for the significant genes in single SNPs regressive analysis in PLINK. This method combined the association *p*-values of SNPs within the same gene (coding sequences± QUOTE _D5kb flanking regions) to calculate a gene-based *p*-value. This gene-based test accounted for gene size and LD pattern. Each SNP in the gene set was searched for its possible SNPs in LD (*r* ^2^=0.5). Standard single SNP QT analysis was then performed. All ‘independent’ SNPs (*r* ^2^=0.5) with *p*-values below 0.05 were selected in the set. The best SNP was selected first and subsequent SNPs were selected in the order of decreasing statistical significance after removing SNPs in LD with previously selected SNPs. The statistical parameters of each SNP subset were calculated as the mean of these single SNP statistics. The data set was further permuted by 10,000 times, keeping LD between SNPs constant, which was also called as permuting trait. The empirical *p*-value for set (empirical *p* value EMP1) was the number of times that the permuted set statistically exceeded the original one of the set. The empirical *p* values were corrected for the multiple SNPs within a set (http://pngu.mgh.harvard.edu/~purcell/plink/anal.shtml#set).

## Results

### Demographic data and Clinical characteristics

In present study, there were 74 patients with schizophrenia (37 males and 37 females) and 51 healthy controls (28 males and 23 females). All patients with schizophrenia were first-episode and treatment-naïve by the time of entering the study. Table 1 represents the demographic and clinical characteristics of all the participants (see Table 1). There were no significant differences in mean age (t=1.29; *p*=0.20), gender distribution (Pearson’s χ^2^=0.29; *p*=0.56) and mean years of education (t=1.44; *p*=0.15) between patients with schizophrenia and normal controls. The mean duration of disease in patients was 9.91 (SD =6.21, range 3-11.2) months and the mean age of onset was 23.67 (SD = 8.56, range 16-35.2) years old when they were recruited. The average of total PANSS score of the patient group was 94.54 (SD =15.00), suggesting that patients were in the acute phases of the disease.

**Table 1:** Clinical and demographic Summaries.

Demographic characteristics	Patients			Controls		p value
Number of subjects	74			51		-----
Gender (male/female)	37/37			28/23		0.59
Mean age (SD)	24.41 (8.00)	Range: [16-49]		26.29 (8.14)	Range: [16-49]	0.20
Education year	12.30 (2.93)	Range: [5-18]		13.06 (2.85)	Range: [5-20]	0.15
Age at onset, year	23.87 (8.56)	Range: [16.5-35.2]				
Duration of illness, Month	9.91 (6.21)	Range: [3-13.2]				
PANSSPANs-P	26.56 (6.00)					
PANSSPANs-N	19.06 (8.00)					
PANSSPANs-G	48.90 (9.00)					
PANSSPANs-T	94.54 (15.00)					
GAF	28 (10)					

Abbreviations: PANSS-P, PANSS subscales for positive symptoms; PANSS-N, PANSS subscales for negative symptoms; PANSS-G, PANSS subscales for general psychopathological symptoms; GAF, global assessment of functioning Scale.

### Reduced GM volume in patients with schizophrenia

By using SPM Anatomy Toolbox, we found that GM volumes in three brain areas including left hOC3v in the collateral sulcus of visual cortex (hOC3vL), left cerebellar vermis lobule 10 (vermisL10) and right cerebellar vermis lobule 10 (vermisR10) were significantly reduced in patients with schizophrenia (See Figure S1). Furthermore, the correlation analysis showed that the correlative coefficient between vermisL10 and vermisR10 was 0.91(*p*=0.001), the correlative coefficient between vermisL10 and hOC3vL was 0.45(*p*=0.02) and the correlative coefficient between vermisL10 and hOC3vL was 0.43(*p*=0.04), respectively.

### Genetic analysis of QT

**Figure 2:**
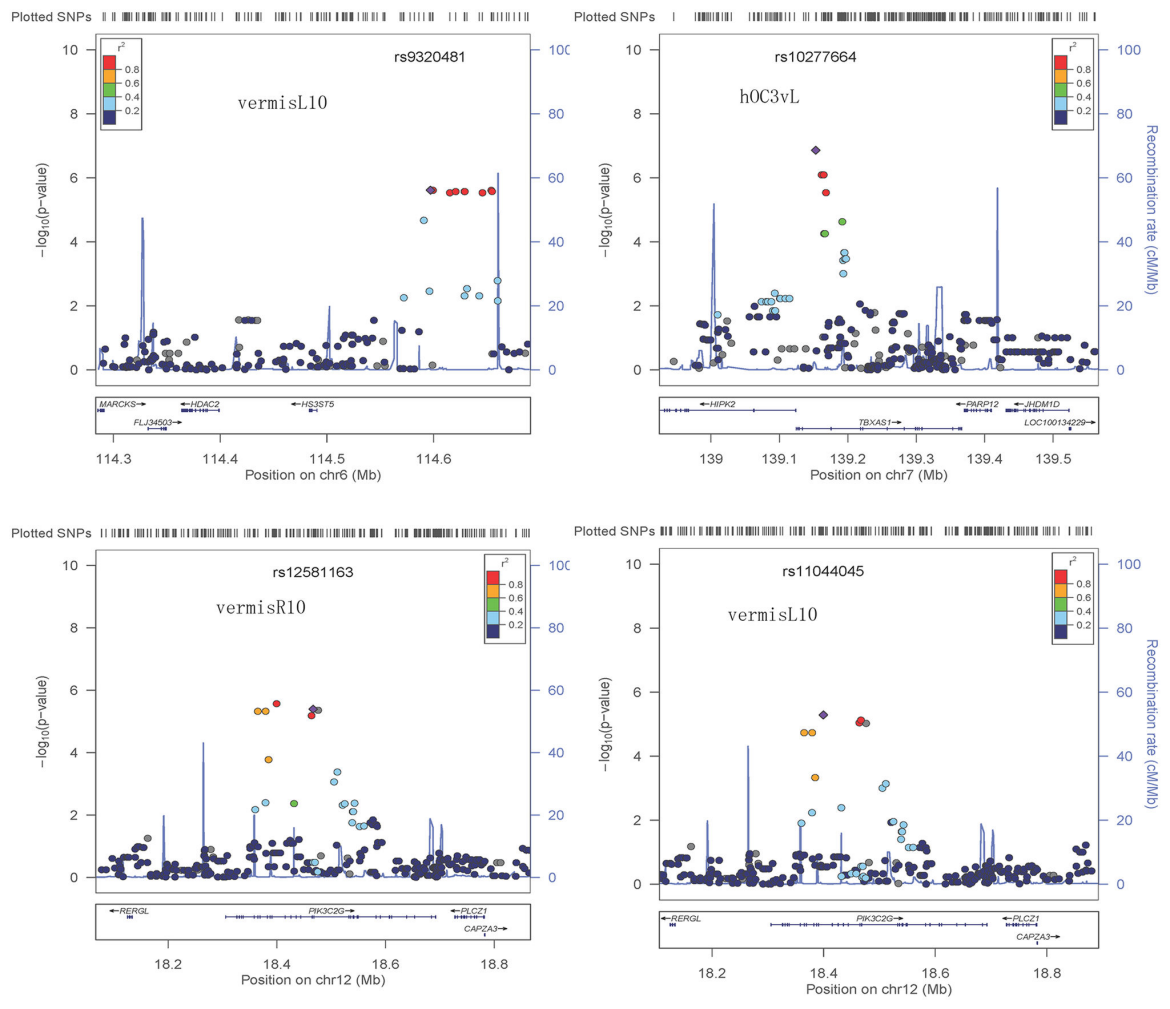
Significance of GWAS Genotyped and imputed SNPs of the 400kb region in chromosomal region of TBXAS1, PIK3C2G and HS3ST5. A, B, C and D are the results of association analysis of vermisL10, hOC3VL, vermisR10 as QT, respectively. Figure 2 was adapted from LocusZoom [49] output.

**Figure 3:**
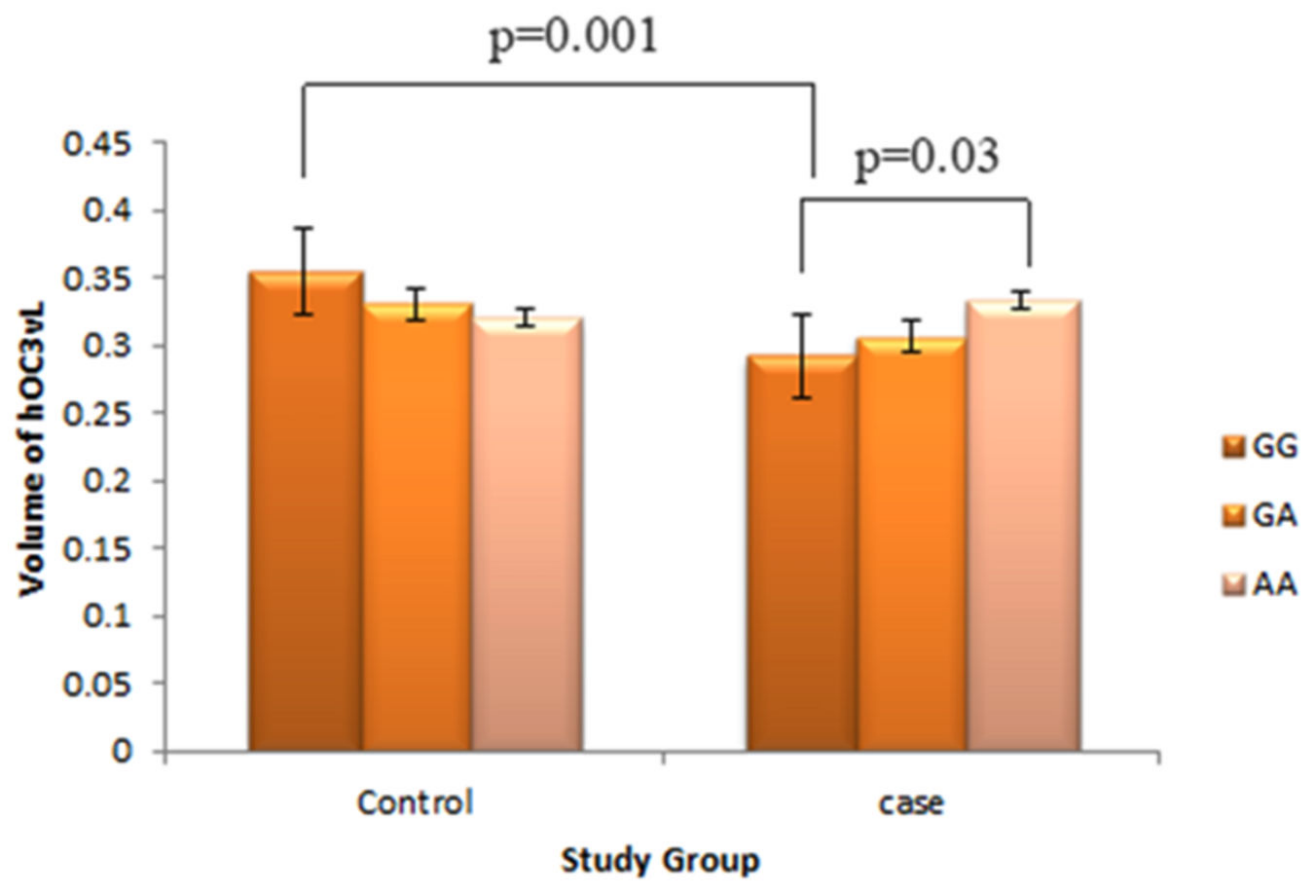
The comparison of the GM volume of hOC3vL of 3 genotypes in cases and controls, respectively. The reduced volume of hOC3vl is associated with the interaction of diagnosis x rs10277664 (*p*<1.38×10^-7^). And the significant difference was detected between the individuals with GG and AA only in case group only (*p*<0.03).

After quality control and genotype imputation, the final dataset consisted of 1,983,054 SNPs from 125 individuals. The genomic inflation factors of 3 endophenotyps after PCA adjustment (λ) were 1.025, 0.991, 1.021 and 1.018 respectively, indicating the absence of major population stratification (see Figure S3). For the interaction terms of SNP x diagnosis, using the criteria that requires at least 2 SNPs within a gene or intergenic region with the *p* value <10^-6^, we identified a number of SNPs from three genes associated significantly with GM volumes in above three brain areas, respectively. As shown in Table 2, 3 SNPs (2 typed and 1 imputed) on the TBXAS1 gene were associated with the GM volume of hOC3vL. 4 SNPs (2 typed and 2 imputed) on the PIK3C2G gene were associated with the GM volume of vermisR10 and vermisL10, with additional 2 SNPs (1 typed and 1 imputed) on PIK3C2G associated only with the GM volume of vermisL10. 9 SNPs (4 typed and 5 imputed) on the HS3ST5 gene were associated with the GM volume of vermisR10. The regional SNPs LD maps of in chromosomal region of TBXAS1, PIK3C2G and HS3ST5 were shown in Figure 2. Moreover, as shown in Figure 3, there were close correlations amongst the SNPs inside each gene. Also, correlations could be found between typed and imputed SNPs.

**Table 2:** GWAS results of significant interaction (SNPs x diagnosis) of 3 candidate endophenotypes.

Chr.	SNPs	Location(bp)	GENE	Type	β	p-value	Traits	SnpType
6	rs9320482	114599292	HS3ST5	Intergenic	0.043	1.14E-06	vermisL10	Typed
6	rs9374441	114620555	HS3ST5	Intergenic	0.04096	8.25E-06	vermisL10	Typed
6	rs9320486	114645676	HS3ST5	Intergenic	0.04201	3.19E-06	vermisL10	Typed
6	rs7746324	114653942	HS3ST5	Intergenic	0.04225	2.74E-06	vermisL10	Typed
6	rs9488343	114654674	HS3ST5	Intergenic	0.04365	7.04E-07	vermisL10	Imputed
6	rs9488340	114615168	HS3ST5	Intergenic	0.04376	8.20E-07	vermisL10	Imputed
6	rs6936717	114628775	HS3ST5	Intergenic	0.0441	8.46E-07	vermisL10	Imputed
6	rs9400701	114629091	HS3ST5	Intergenic	0.0441	8.46E-07	vermisL10	Imputed
6	rs9320481	114596974	HS3ST5	Intergenic	0.04298	1.04E-06	vermisL10	Imputed
7	rs10277664	139153049	TBXAS1	Intronic	-0.04315	1.38E-07	hOC3vL	Typed
7	rs1107952	139164674	TBXAS1	Intronic	-0.04051	8.04E-07	hOC3vL	Typed
7	rs10233386	139161685	TBXAS1	Intronic	-0.04054	8.01E-07	hOC3vL	Imputed
12	rs10770359	18463869	PIK3C2G	Intronic	-0.04721	9.02E-06	vermisR10	Typed
12	rs12581163	18466821	PIK3C2G	Intronic	-0.04763	5.56E-06	vermisR10	Typed
12	rs11044045	18399519	PIK3C2G	Intronic	-0.05253	5.15E-06	vermisR10	Imputed
12	rs11044106	18475943	PIK3C2G	Intronic	-0.04707	9.39E-06	vermisR10	Imputed
12	rs11044045	18399519	PIK3C2G	Intronic	-0.04449	2.69E-06	vermisL10	Imputed
12	rs11044106	18475943	PIK3C2G	Intronic	-0.04024	4.36E-06	vermisL10	Imputed
12	rs12580062	18379331	PIK3C2G	Intronic	-0.04192	4.71E-06	vermisL10	Imputed
12	rs11044026	18364996	PIK3C2G	Intronic	-0.04184	5.45E-06	vermisL10	Typed
12	rs10770359	18463869	PIK3C2G	Intronic	-0.03962	6.50E-06	vermisL10	Typed
12	rs12581163	18466821	PIK3C2G	Intronic	-0.04053	2.70E-06	vermisL10	Typed

The effect per minor allele on GM volume of above three brain areas was relatively small, with β approximately 4% (Table 2). The SNP effect of the minor allele in TBXAS1 and PIK3C2G genes were consistent with the reduced GM volume of hOC3vL and bilateral cerebellar vermis lobule 10, respectively. However, the effect of the minor allele in HS3ST5 gene was correlated with the increased GM volume of vermisR10.

Furthermore, we compared the GM volumes of three brain areas according to genotypes of each significant SNP in table 2 in cases and controls, respectively. We found that patients with homozygous GG of rs10277664 in TBXAS1 gene have reduced GM volume of hOC3vL while compared to healthy controls (*p*<0.001), but there were no significant difference for subjects with heterozygous (AG) or homozygotes (AA) between patients and controls. We also found that there was a significant smaller GM volume of hOC3vL in individuals with genotype GG of rs10277664 in TBXAS1 gene than individuals with genotype AA in patients only (*p*<0.03) (see Figure 3).

We also used set-based analysis to obtain the combined association *p*-values of SNPs within the same gene for each QT. Table S1 showed gene-wise *p* values, number of SNPs in set, total number of SNPs below p-value threshold, and number of significant SNPs passing LD-criterion.

## Discussion

In present study, we used the SPM Anatomy Toolbox to obtain anatomical ROIs volumes and detected significant differences of the GM volumes in hOC3vL, vermisL10 and vermisR10 between patients with schizophrenia and healthy controls. The GM volume in the three regions was found to be correlated with each other, which may imply the role of same or shared genetic factors to these deficits in these brain regions. Furthermore, by using GM volume from these 3 brain areas as QT, we performed a GWAS on 74 patients with schizophrenia and 51 controls from a Han Chinese population and identified a number of SNPs from three genes or chromosomal regions (TBXAS1, PIK3C2G and HS3ST5) were associated with changed GM volumes of hOC3vL, vermisL10 and vermisR10. The fact that more than 2 SNPs on the genotyping chip exhibited association and they were in strong LD confirmed that the significant association was not likely to result from a genotyping artifact. Moreover, imputed SNPs were identified to be significantly associated with candidate endophenotypes, which further confirmed that the associations detected in our study were genuine.

The hOC3v area is located in the collateral sulcus of visual cortex and represents the anatomical substrates of the functionally defined areas of VP/V3v [21]. Previous studies on neurocognitive deficits of schizophrenia have been mainly focused on executive function, attention and working memory with less attention on perceptual processing. Studies have shown that perceptual deficits are one of core features of clinical manifestations in patients with schizophrenia [22-26]. The lower local sulcal index in the left collateral sulcus were detected in patients with early-onset schizophrenia by Penttilä et al. [27]. The present study adds new evidence for the deficit of visual processing in patients with schizophrenia, which support the hypothesis of dysfunction within low-level visual pathways involving thalamocortical radiations [22,28]. On the other hand, cerebellar vermis consists of ten lobules (lobule I-X) [29] and some studies found that the GM deficits in first-episode schizophrenia were more prominent in the right lobule III and IX of cerebellar vermis while compared to healthy controls [30]. To the best of our knowledge, the present study is the first one to identify the GM deficits in bilateral lobule X in patients with schizophrenia.

Three genes (TBXAS1, PIK3C2G and HS3ST5) have been implicated with the GM volume changes of cortex in schizophrenia, particularly in the collateral sulcus of visual cortex and cerebellar vermis lobule 10. PIK3C2G gene is located to 12p12 and it encodes the protein which belongs to the phosphoinositide 3-kinase (PI3K) family. Previous studies suggested that the PI3K plays important role in the myelin-signaling pathway, involved in cell proliferation, oncogenic transformation, cell survival, cell migration and intracellular protein trafficking. PI3K also involved in the prosurvival effects of BDNF in the SH-SY5Y neuroblastoma cell line, as well as in the protective effects of BDNF against cortical neuronal death. Jungerius et al. found a weak but significant association between PIK3C2G gene and schizophrenia [31]. HS3ST5 gene is located in 6q21 and encodes a protein that belongs to a group of heparan sulfate 3-O-sulfotransferases (EC 2.8.2.23) and transfers sulfate from 3-prime-phosphoadenosine 5-prime phosphosulfate (PAPS) to heparan sulfate and heparin. The product of HS3ST5 gene, which is a member of the human 3-OST family, regulates heparan sulfate binding to AT III and Gd. A study has revealed that 3-OST-5 was highly expressed in fetal brain, followed by adult brain and spinal cord. Its level is very low or undetectable in other tissues [32]. In addition, this chromosome region (6q21) has long been of interest for studying schizophrenia although replicating positive linkage and association in schizophrenia are difficult [33]. TBXAS1 gene is located to 7q34-q35 and has been related with the physiopathology of a number of diseases including cerebral infarction, myocardial infarction and stroke [34,35]. It belongs to the cytochrome P450 superfamily. Alternative spliced transcript variants encoding different isoforms have been found for this gene. The cytochrome P450 proteins are monooxygenases that catalyze reactions involved in drug metabolism and the synthesis of cholesterol, steroids and other lipids. This endoplasmic reticulum membrane protein catalyzes the conversion of prostglandin H2 to thromboxane A2, a potent vasoconstrictor and an inducer of platelet aggregation.

It should be noted that the majority of SNPs on the Illumina Infinium HumanHap 660 BeadArray are tagging SNPs as surrogate makers to represent a given small chromosome region. The association identified in present study suggested evidence that areas of three genes (TBXAS1, PIK3C2G and HS3ST5) or related chromosome regions harbor susceptibility allele for schizophrenia with GM volume as QT. However, it is likely that the causal SNPs for the disorder are just located in close proximity to these positive SNPs identified in current study and requires confirmation in independent samples. Future studies should consider sequencing these genomic regions in a larger cohort of patients with schizophrenia to identify causal genetic variants for schizophrenia. It will also be useful to explore the biological functions of these genes for the disease.

By using the case-control approach, previous GWAS of schizophrenia have detected several noteworthy candidate genes [4,5]. However there has been little consistency in those findings. In current study, the linear model with interaction of diagnosis x SNPs has been used with the advantageous to detect the effects of different genotypes between case and control in QT analysis. In order to exclude the false positive in the QT analyses in the interaction term model, we chose a threshold of p value <10^-6^ for at least 2 independent SNPs within a gene/intergenic region in each QT analysis. Moreover, this study has another advantage by including the first-episode treatment-naïve patients whereas most previous studies of QT GWAS included chronic and long-term drugs-taking patients [7,36]. The latter could disguise the effects of some confounding factors, such as antipsychotic medications, duration of illness, hospitalization and so on. Ho et al. found that antipsychotics had a subtle but measurable influence on brain tissue losses over time in a long-term longitudinal study of 211 patients over 7-14 years follow up [37]. More extensive structural brain abnormalities were found in chronic patients with schizophrenia that may reflect the progressive nature of this condition compared to first-episode patients with schizophrenia. Thus the brain deficits in early stage could reflect the features of neurodevelopmental deficits of schizophrenia. With inconsistent with our finding, Hu et al. and Leung et al. found that grey matter deficits in frontal, temporal, insular, striatal, posterior cingulate, and cerebellar for first-episode drug naïve schizophrenic patients [38,39]. We should acknowledge that it is difficult to detect the similar brain regions with deficits between different studies. And the difference could be due to lots of reasons, especially methodology. In our study, we used the SPM Anatomy Toolbox to obtain ROI regions which was a finer segmentation for whole brain, and could partly account for the difference in results. In addition, sample size and race of participants could partly contribute to the difference too.

It should be acknowledged that our study still has some limitations. First, the sample size was not big enough for GWAs. Second, this study needs to confirm by independent replications, especially in non-Chinese samples. Third, the SNPs detected were in non-encoding regions of the genes, so further functional analyses are needed for these genes. Finally, since environment factors, such as city living and urban upbringing [40], play an important role in brain development and risk of schizophrenia, it will be better for this study to include environmental factors and examine their roles in the pathogenesis of schizophrenia.

In summary, through the GWAS approach combined with GM volume as QTs, we identified some novel susceptibility loci for schizophrenia on 3 genes/intergenic regions, namely TBXAS1, PIK3C2G and HS3ST5, as potential risk factors for schizophrenia in first-episode treatment-naïve patients with schizophrenia and controls. The present study also highlighted the usefulness of endophenotype in exploring the pathogenesis of neuropsychiatric diseases such as schizophrenia although further independent replication studies are warranted in the future.

## Supporting Information

Figure S1 **The plot of the first two principal components of 74 patients with schizophrenia and 51 controls as output from EIGENSOFT (Price, 2006).** Each data point represents an individual.
(TIF)

Figure S2 **Three ROIs that were detected between 74 patients with schizophrenia and 51 healthy controls (HOC3VL, vermisL10 and vermisR10) using SPM anatomy toolbox.**
(TIF)

Figure S3 **QQ plot of p-values, after adjustment by principal components derived from EIGENSTRAT.**
(TIF)

Table S1 **Set-based test results of TBXAS1, PIK3C2G and HS3ST5.**
(DOCX)

## References

[1] van Os J, Kapur S (2009) Schizophrenia. Lancet 374: 635-645. doi:10.1016/S0140-6736(09)60995-8. PubMed: 19700006. 19700006 10.1016/S0140-6736(09)60995-8

[2] Gottesman II, McGuffin P, Farmer AE (1987) Clinical genetics as clues to the ¡°real¡± genetics of schizophrenia (a decade of modest gains while playing for time). Schizophr Bull 13: 23-47. doi:10.1093/schbul/13.1.23. PubMed: 3474774. 3474774 10.1093/schbul/13.1.23

[3] McGuffin P, Gottesman II (1999) Risk factors for schizophrenia. N Engl J Med 341: 370-371; author reply: 372 doi:10.1056/NEJM199907293410513. PubMed: 10428662. 10428662

[4] Shi Y, Li Z, Xu Q, Wang T, Li T et al. (2011) Common variants on 8p12 and 1q24.2 confer risk of schizophrenia. Nat Genet 43: 1224-1227. doi:10.1038/ng.980. PubMed: 22037555. 22037555 10.1038/ng.980 PMC3773910

[5] Yue WH, Wang HF, Sun LD, Tang FL, Liu ZH et al. (2011) Genome-wide association study identifies a susceptibility locus for schizophrenia in Han Chinese at 11p11.2. Nat Genet 43: 1228-1231. doi:10.1038/ng.979. PubMed: 22037552. 22037552 10.1038/ng.979

[6] Cardon LR, Craddock N, Deloukas P, Duncanson A, Kwiatkowski DP et al. (2007) Genome-wide association study of 14,000 cases of seven common diseases and 3,000 shared controls. Nature 447: 661-678. doi:10.1038/nature05911. PubMed: 17554300. 17554300 10.1038/nature05911 PMC2719288

[7] Potkin SG, Turner JA, Guffanti G, Lakatos A, Fallon JH et al. (2009) A genome-wide association study of schizophrenia using brain activation as a quantitative phenotype. Schizophr Bull 35: 96-108. doi:10.1093/schbul/sbn155. PubMed: 19023125. 19023125 10.1093/schbul/sbn155 PMC2643953

[8] Gottesman II, Gould TD (2003) The endophenotype concept in psychiatry: etymology and strategic intentions. Am J Psychiatry 160: 636-645. doi:10.1176/appi.ajp.160.4.636. PubMed: 12668349. 12668349 10.1176/appi.ajp.160.4.636

[9] Fornito A, Yücel M, Patti J, Wood SJ, Pantelis C (2009) Mapping grey matter reductions in schizophrenia: an anatomical likelihood estimation analysis of voxel-based morphometry studies. Schizophr Res 108: 104-113. doi:10.1016/j.schres.2008.12.011. PubMed: 19157788. 19157788 10.1016/j.schres.2008.12.011

[10] Rijsdijk FV, van Haren NE, Picchioni MM, McDonald C, Toulopoulou T et al. (2005) Brain MRI abnormalities in schizophrenia: same genes or same environment? Psychol Med 35: 1399-1409. doi:10.1017/S0033291705005167. PubMed: 16164764. 16164764 10.1017/S0033291705005167

[11] Miyata J, Hirao K, Namiki C, Fujiwara H, Shimizu M et al. (2009) Reduced white matter integrity correlated with cortico-subcortical gray matter deficits in schizophrenia. Schizophr Res 111: 78-85. doi:10.1016/j.schres.2009.03.010. PubMed: 19361957. 19361957 10.1016/j.schres.2009.03.010

[12] Voets NL, Hough MG, Douaud G, Matthews PM, James A et al. (2008) Evidence for abnormalities of cortical development in adolescent-onset schizophrenia. Neuroimage 43: 665-675. doi:10.1016/j.neuroimage.2008.08.013. PubMed: 18793730. 18793730 10.1016/j.neuroimage.2008.08.013

[13] Dutt A, McDonald C, Dempster E, Prata D, Shaikh M et al. (2009) The effect of COMT, BDNF, 5-HTT, NRG1 and DTNBP1 genes on hippocampal and lateral ventricular volume in psychosis. Psychol Med 39: 1783-1797. doi:10.1017/S0033291709990316. PubMed: 19573260. 19573260 10.1017/S0033291709990316

[14] Brun CC, Leporé N, Pennec X, Lee AD, Barysheva M et al. (2009) Mapping the regional influence of genetics on brain structure variability--a tensor-based morphometry study. Neuroimage 48: 37-49. doi:10.1016/j.neuroimage.2009.05.022. PubMed: 19446645. 19446645 10.1016/j.neuroimage.2009.05.022 PMC2859973

[15] Matsuo K, Walss-Bass C, Nery FG, Nicoletti MA, Hatch JP et al. (2009) Neuronal correlates of brain-derived neurotrophic factor Val66Met polymorphism and morphometric abnormalities in bipolar disorder. Neuropsychopharmacology 34: 1904-1913. doi:10.1038/npp.2009.23. PubMed: 19295510. 19295510 10.1038/npp.2009.23

[16] Stein JL, Hua X, Lee S, Ho AJ, Leow AD et al. (2010) Voxelwise genome-wide association study (vGWAS). Neuroimage 53: 1160-1174. doi:10.1016/j.neuroimage.2010.02.032. PubMed: 20171287. 20171287 10.1016/j.neuroimage.2010.02.032 PMC2900429

[17] Stein JL, Medland SE, Vasquez AA, Hibar DP, Senstad RE et al. (2012) Identification of common variants associated with human hippocampal and intracranial volumes. Nat Genet 44: 552-561. doi:10.1038/ng.2250. PubMed: 22504417. 22504417 10.1038/ng.2250 PMC3635491

[18] Eickhoff SB, Stephan KE, Mohlberg H, Grefkes C, Fink GR et al. (2005) A new SPM toolbox for combining probabilistic cytoarchitectonic maps and functional imaging data. NeuroImage 25: 1325-1335. doi:10.1016/j.neuroimage.2004.12.034. PubMed: 15850749. 15850749 10.1016/j.neuroimage.2004.12.034

[19] Eickhoff SB, Heim S, Zilles K, Amunts K (2006) Testing anatomically specified hypotheses in functional imaging using cytoarchitectonic maps. NeuroImage 32: 570-582. doi:10.1016/j.neuroimage.2006.04.204. PubMed: 16781166. 16781166 10.1016/j.neuroimage.2006.04.204

[20] Eickhoff SB, Paus T, Caspers S, Grosbras MH, Evans AC et al. (2007) Assignment of functional activations to probabilistic cytoarchitectonic areas revisited. NeuroImage 36: 511-521. doi:10.1016/j.neuroimage.2007.03.060. PubMed: 17499520. 17499520 10.1016/j.neuroimage.2007.03.060

[21] Rottschy C, Eickhoff SB, Schleicher A, Mohlberg H, Kujovic M et al. (2007) Ventral visual cortex in humans: cytoarchitectonic mapping of two extrastriate areas. Hum Brain Mapp 28: 1045-1059. doi:10.1002/hbm.20348. PubMed: 17266106. 17266106 10.1002/hbm.20348 PMC6871378

[22] Rametti G, Junqué C, Bartrés-Faz D, Zubiaurre-Elorza L, Catalán R et al. (2010) Anterior cingulate and paracingulate sulci morphology in patients with schizophrenia. Schizophr Res 121: 66-74. doi:10.1016/j.schres.2010.05.016. PubMed: 20547448. 20547448 10.1016/j.schres.2010.05.016

[23] Kakeda S, Korogi Y (2010) The efficacy of a voxel-based morphometry on the analysis of imaging in schizophrenia, temporal lobe epilepsy, and Alzheimer’s disease/mild cognitive impairment: a review. Neuroradiology 52: 711-721. doi:10.1007/s00234-010-0717-2. PubMed: 20495793. 20495793 10.1007/s00234-010-0717-2

[24] Pomarol-Clotet E, Canales-Rodríguez EJ, Salvador R, Sarró S, Gomar JJ et al. (2010) Medial prefrontal cortex pathology in schizophrenia as revealed by convergent findings from multimodal imaging. Mol Psychiatry 15: 823-830. PubMed: 20065955. 20065955 10.1038/mp.2009.146 PMC2927029

[25] Takao H, Abe O, Yamasue H, Aoki S, Kasai K et al. (2010) Cerebral asymmetry in patients with schizophrenia: a voxel-based morphometry (VBM) and diffusion tensor imaging (DTI) study. J Magn Reson Imaging 31: 221-226. doi:10.1002/jmri.22017. PubMed: 20027592. 20027592 10.1002/jmri.22017

[26] Jacobson S, Kelleher I, Harley M, Murtagh A, Clarke M et al. (2010) Structural and functional brain correlates of subclinical psychotic symptoms in 11-13 year old schoolchildren. Neuroimage 49: 1875-1885. doi:10.1016/j.neuroimage.2009.09.015. PubMed: 19770054. 19770054 10.1016/j.neuroimage.2009.09.015

[27] Penttilä J, Paillére-Martinot ML, Martinot JL, Mangin JF, Burke L et al. (2008) Global and temporal cortical folding in patients with early-onset schizophrenia. J Am Acad Child Adolesc Psychiatry 47: 1125-1132. doi:10.1097/CHI.0b013e3181825aa7. PubMed: 18725863. 18725863 10.1097/CHI.0b013e3181825aa7

[28] Turcan S, Rohle D, Goenka A, Walsh LA, Fang F et al. (2012) IDH1 mutation is sufficient to establish the glioma hypermethylator phenotype. Nature 483: 479-483. doi:10.1038/nature10866. PubMed: 22343889. 22343889 10.1038/nature10866 PMC3351699

[29] Diedrichsen J, Balsters JH, Flavell J, Cussans E, Ramnani N (2009) A probabilistic MR atlas of the human cerebellum. Neuroimage 46: 39-46. doi:10.1016/j.neuroimage.2009.01.045. PubMed: 19457380. 19457380 10.1016/j.neuroimage.2009.01.045

[30] Rasser PE, Schall U, Peck G, Cohen M, Johnston P et al. (2010) Cerebellar grey matter deficits in first-episode schizophrenia mapped using cortical pattern matching. NeuroImage 53: 1175-1180. doi:10.1016/j.neuroimage.2010.07.018. PubMed: 20633666. 20633666 10.1016/j.neuroimage.2010.07.018

[31] Jungerius BJ, Hoogendoorn ML, Bakker SC, Van’t Slot R, Bardoel AF et al. (2008) An association screen of myelin-related genes implicates the chromosome 22q11 PIK4CA gene in schizophrenia. Mol Psychiatry 13: 1060-1068. PubMed: 17893707. 17893707 10.1038/sj.mp.4002080

[32] Mochizuki H, Yoshida K, Gotoh M, Sugioka S, Kikuchi N et al. (2003) Characterization of a heparan sulfate 3-O-sulfotransferase-5, an enzyme synthesizing a tetrasulfated disaccharide. J Biol Chem 278: 26780–26787. doi:10.1074/jbc.M301861200. PubMed: 12740361. 12740361 10.1074/jbc.M301861200

[33] Owen MJ, Williams NM, O’Donovan MC (2004) The molecular genetics of schizophrenia: new findings promise new insights. Mol Psychiatry 9: 14-27. doi:10.1038/sj.mp.4001444. PubMed: 14581932. 14581932 10.1038/sj.mp.4001444

[34] Tsujita Y, Kinoshita M, Tanabe T, Iwai N (2000) Role of a genetic variation in the promoter of human thromboxane synthase gene in myocardial infarction. Atherosclerosis 153: 261-262. doi:10.1016/S0021-9150(00)00593-1. PubMed: 11184633. 11184633 10.1016/s0021-9150(00)00593-1

[35] Park SA, Park BL, Park JH, Lee TK, Sung KB et al. (2009) Association of polymorphisms in thromboxane A2 receptor and thromboxane A synthase 1 with cerebral infarction in a Korean population. Population 42: 200-205. 10.5483/bmbrep.2009.42.4.200 19403042

[36] Maloku E, Kadriu B, Zhubi A, Dong E, Pibiri F et al. (2011) Selective alpha4beta2 nicotinic acetylcholine receptor agonists target epigenetic mechanisms in cortical GABAergic neurons. Neuropsychopharmacology 36: 1366-1374. PubMed: 21368748. 21368748 10.1038/npp.2011.21 PMC3096806

[37] Ho BC, Andreasen NC, Ziebell S, Pierson R, Magnotta V (2011) Long-term antipsychotic treatment and brain volumes: a longitudinal study of first-episode schizophrenia. Arch Gen Psychiatry 68: 128-137. doi:10.1001/archgenpsychiatry.2010.199. PubMed: 21300943. 21300943 10.1001/archgenpsychiatry.2010.199 PMC3476840

[38] Leung M, Cheung C, Yu K, Yip B, Sham P et al. (2011) Gray matter in first-episode schizophrenia before and after antipsychotic drug treatment. Anatomical likelihood estimation meta-analyses with sample size weighting. Schizophr Bull 37: 199-211. doi:10.1093/schbul/sbp099. PubMed: 19759093. 19759093 10.1093/schbul/sbp099 PMC3004197

[39] Hu M, Li J, Eyler L, Guo X, Wei Q et al. (2013) Decreased left middle temporal gyrus volume in antipsychotic drug-naive, first-episode schizophrenia patients and their healthy unaffected siblings. Schizophr Res, 144: 37–42. PubMed: 23360727. 23360727 10.1016/j.schres.2012.12.018

[40] Lederbogen F, Kirsch P, Haddad L, Streit F, Tost H et al. (2011) City living and urban upbringing affect neural social stress processing in humans. Nature 474: 498-501. PubMed: 21697947. 21697947 10.1038/nature10190

[41] Rorden C, Brett M (2000) Stereotaxic display of brain lesions. Behav Neurol 12: 191-200. PubMed: 11568431. 11568431 10.1155/2000/421719

[42] Smith SM (2002) Fast robust automated brain extraction. Hum Brain Mapp 17: 143-155. doi:10.1002/hbm.10062. PubMed: 12391568. 12391568 10.1002/hbm.10062 PMC6871816

[43] Ashburner J, Friston KJ (2005) Unified segmentation. NeuroImage 26: 839-851. doi:10.1016/j.neuroimage.2005.02.018. PubMed: 15955494. 15955494 10.1016/j.neuroimage.2005.02.018

[44] Li MX, Jiang L, Kao PY, Sham PC, Song YQ (2009) IGG3: a tool to rapidly integrate large genotype datasets for whole-genome imputation and individual-level meta-analysis. Bioinformatics 25: 1449-1450. doi:10.1093/bioinformatics/btp183. PubMed: 19346322. 19346322 10.1093/bioinformatics/btp183

[45] Price AL, Patterson NJ, Plenge RM, Weinblatt ME, Shadick NA et al. (2006) Principal components analysis corrects for stratification in genome-wide association studies. Nat Genet 38: 904-909. doi:10.1038/ng1847. PubMed: 16862161. 16862161 10.1038/ng1847

[46] Price AL, Weale ME, Patterson N, Myers SR, Need AC et al. (2008) Long-range LD can confound genome scans in admixed populations. Am J Hum Genet 83: 132-135; author reply: 135-139 18606306 10.1016/j.ajhg.2008.06.005 PMC2443852

[47] Brown GG, Lee JS, Strigo IA, Caligiuri MP, Meloy MJ et al. (2011) Voxel-based morphometry of patients with schizophrenia or bipolar I disorder: a matched control study. Psychiatry Res 194: 149-156. doi:10.1016/j.pscychresns.2011.05.005. PubMed: 21924872. 21924872 10.1016/j.pscychresns.2011.05.005 PMC3196272

[48] Purcell S, Neale B, Todd-Brown K, Thomas L, Ferreira MAR et al. (2007) PLINK: a tool set for whole-genome association and population-based linkage analyses. Am J Hum Genet 81: 559-575. doi:10.1086/519795. PubMed: 17701901. 17701901 10.1086/519795 PMC1950838

[49] Watson DR, Anderson JM, Bai F, Barrett SL, McGinnity TM et al. (2012) A voxel based morphometry study investigating brain structural changes in first episode psychosis. Behav Brain Res 227: 91-99. doi:10.1016/j.bbr.2011.10.034. PubMed: 22056751. 22056751 10.1016/j.bbr.2011.10.034

