# Correction: Genome-Wide Association Analysis with Gray Matter Volume as a Quantitative Phenotype in First-Episode Treatment-Naïve Patients with Schizophrenia

**DOI:** 10.1371/journal.pone.0122945

**Published:** 2015-04-07

**Authors:** The PLOS ONE Staff

There is an error in the sixth sentence of the Abstract. The correct sentence is: Reduced GM volumes in three brain areas including right hOC3v in the collateral sulcus of visual cortex (hOC3vR), left cerebellar vermis lobule 10 (vermisL10) and right cerebellar vermis lobule 10 (vermisR10) were found in patients with schizophrenia.

There is an error in the first sentence of the Reduced GM volume in patients with schizophrenia subsection of the Results. The correct sentence is: By using SPM Anatomy Toolbox, we found that GM volumes in three brain areas including left hOC3vR in the collateral sulcus of visual cortex (hOC3vL), left cerebellar vermis lobule 10 (vermisL10) and right cerebellar vermis lobule 10 (vermisR10) were significantly reduced in patients with schizophrenia (See Figure S1).

The term hOC3vL appears incorrectly throughout the manuscript. The correct term is hOC3vR.

S2 Fig is incorrect. The complete, correct S2 Fig is:

## Supporting Information

S2 Fig Three ROIs that were detected between 74 patients with schizophrenia and 51 healthy controls (hOC3vR, vermisL10 and vermisR10) using SPM anatomy toolbox. (TIF)
