# Supplementary figures and images for: Genome-Wide Association Analysis with Gray Matter Volume as a Quantitative Phenotype in First-Episode Treatment-Naïve Patients with Schizophrenia
Source: PLoS One. 2013 Sep 24;8(9):e75083. doi: 10.1371/journal.pone.0075083 (PMC3782493; doi:10.1371/journal.pone.0075083)

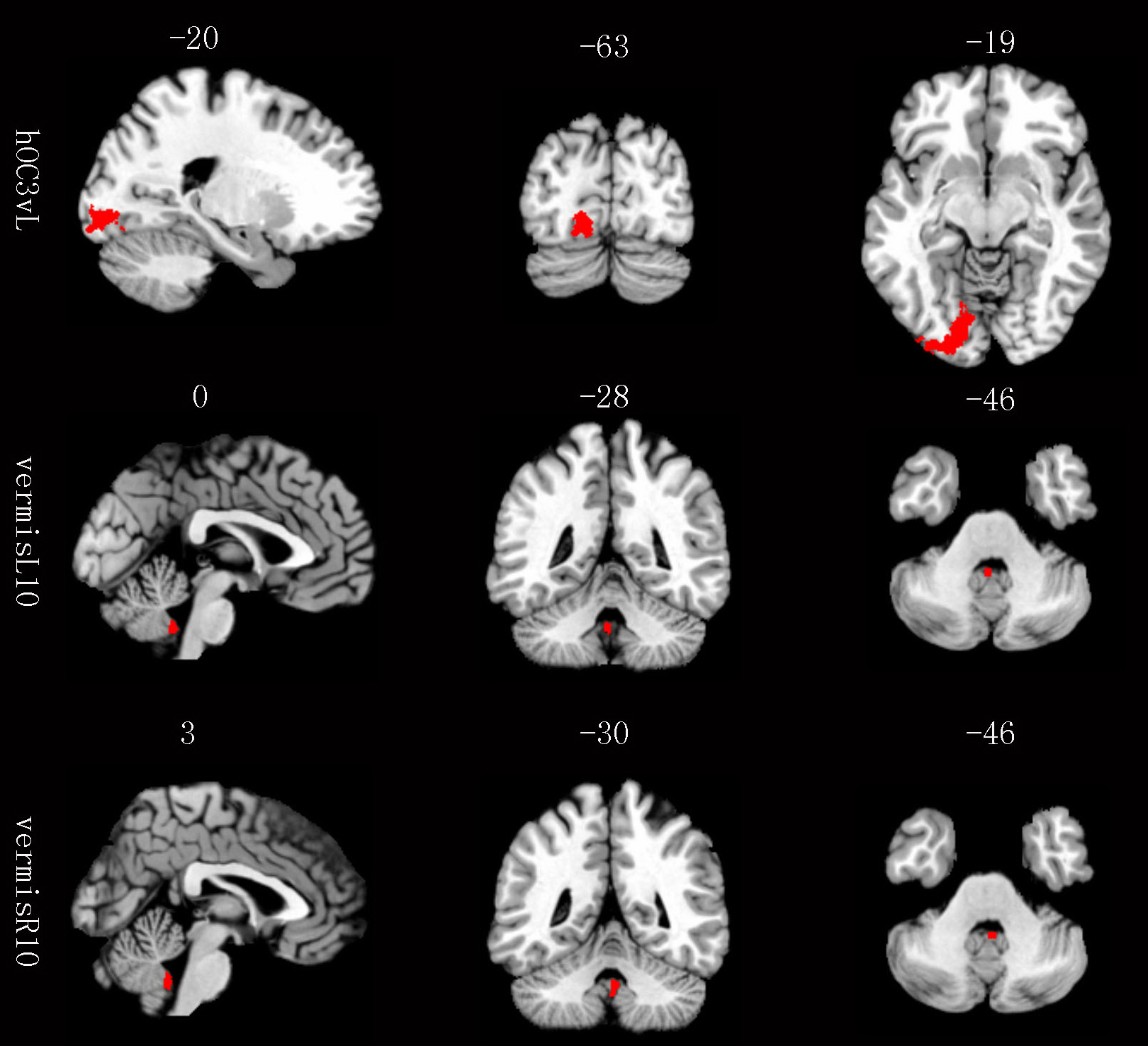

Supplement: Figure S2 — Three ROIs that were detected between 74 patients with schizophrenia and 51 healthy controls (HOC3VL, vermisL10 and vermisR10) using SPM anatomy toolbox. (TIF) [file pone.0075083.s002.tif]

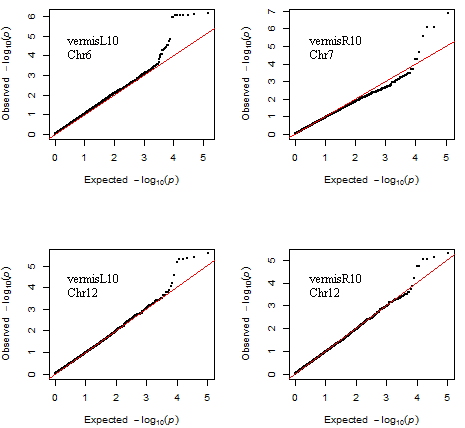

Supplement: Figure S3 — QQ plot of p-values, after adjustment by principal components derived from EIGENSTRAT. (TIF) [file pone.0075083.s003.tif]
