# Supplementary material for: Genome-Wide Association Analysis with Gray Matter Volume as a Quantitative Phenotype in First-Episode Treatment-Naïve Patients with Schizophrenia
Source: PLoS One. 2013 Sep 24;8(9):e75083. doi: 10.1371/journal.pone.0075083 (PMC3782493; doi:10.1371/journal.pone.0075083)
Supplement: Table S1 — Set-based test results of TBXAS1, PIK3C2G and HS3ST5. (DOCX) [file pone.0075083.s004.docx]

**Table S1.** Set-based test results of TBXAS1, PIK3C2G and HS3ST5

| Gene symbol （QT） | p-values (Gene-wise) | Chromosome | Start position | Length (in bp) | NSIG | ISIG | NSNP |
| --- | --- | --- | --- | --- | --- | --- | --- |
| HS3ST5 (vermisL10) | 0.0076 | 6 | 114483442 | 7292 | 15 | 5 | 550 |
| TBXAS1 (hOC3vL) | 0.000599 | 7 | 139175420 | 191051 | 14 | 5 | 240 |
| PIK3C2G (vermis10L) | 0.0034 | 12 | 18305740 | 386877 | 13 | 5 | 236 |
| PIK3C2G (vermisR10) | 0.0157 | 12 | 18305740 | 386877 | 13 | 5 | 236 |

NSIG, Total number of SNPs below p-value threshold

ISIG, Number of significant SNPs also passing LD-criterion

NSNP, Number of SNPs in set
