# Supplementary figures and images for: Correction: Genome-Wide Association Analysis with Gray Matter Volume as a Quantitative Phenotype in First-Episode Treatment-Naïve Patients with Schizophrenia
Source: PLoS One. 2015 Apr 7;10(4):e0122945. doi: 10.1371/journal.pone.0122945 (PMC4388674; doi:10.1371/journal.pone.0122945)

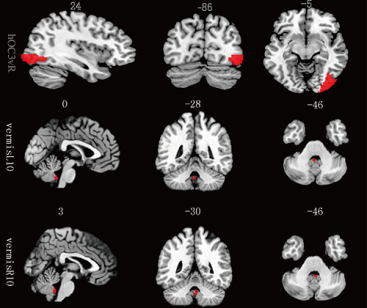

Supplement: S2 Fig — (TIF) [file pone.0122945.s001.tif]
